# Balancing Anterior and Posterior Cruciate Ligaments in Adults

**DOI:** 10.7759/cureus.59683

**Published:** 2024-05-05

**Authors:** Eduard M Cernat, Andrei Neagu, Cezar Betianu, Loredana Sabina Cornelia Manolescu, George Avram, Mark-Edward Pogarasteanu, Adrian Barbilian

**Affiliations:** 1 Department of Clinical Education, Faculty of Medicine, Carol Davila University of Medicine and Pharmacy, Bucharest, ROU; 2 Department of Orthopaedics, Doctor Carol Davila Central Military University Emergency Hospital, Bucharest, ROU; 3 Department of Radiology, Doctor Carol Davila Central Military University Emergency Hospital, Bucharest, ROU; 4 Department of Fundamental Science, Faculty of Midwifery and Nursing, University of Medicine and Pharmacy Carol Davila, Bucharest, ROU

**Keywords:** notch, acl cross sectional area, pcl cross sectional area, posterior cruciate ligament (pcl), anterior cruciate ligament (acl)

## Abstract

Background: The anterior cruciate ligament (ACL) and posterior cruciate ligament (PCL) represent the central pivot of the knee. The balance between these two ligaments impacts the tibiofemoral biomechanics. Each structure is the opposite of the other in terms of anteroposterior translation and rotation.

Aim: The aim of this study was to find a correlation between the cross-sectional area of the ACL and PCL in adults.

Material and methods: Magnetic resonance imaging (MRI) data analysis was conducted by a musculoskeletal radiologist using MRI planes tailored to the study's requirements. In all 62 studied patients, measurements were done according to the protocol.

Results: The study observed three types of intercondylar notches: Type U was identified in 35% (22) of patients, type W in 27% (17), and type A in 37% (23). The median difference between the ACL and PCL areas was found to be statistically significant (p = 0.02). A significant difference in the area of the ACL was detected between Type A and Type U notches (p = 0.02), while no significant differences were found between Type A-W and Type W-U after post hoc corrections (p > 0.05). Additionally, no significant difference was observed in the mean area of the PCL across all three notch types (p = 0.1). In 68% of the cases, the ACL is no less than 60% of the PCL in area, and no more than 120%. The size of ACL and PCL in healthy individuals also depends on other factors like synergistic and antagonistic muscle activities, occupation, and the hip-knee-ankle axis. For example, if the PCL area is 0.79 cm² and the measured structure is round (during a reconstruction a hamstring graft is round), the diameter is 10 mm. A native ACL is, in 68% of the cases, no less than 7.7 mm, and no more than 10.9 mm.

Conclusion: The ACL-PCL size correlation helps in understanding the balance of the central pivot of the knee.

## Introduction

The anterior cruciate ligament (ACL) and posterior cruciate ligament (PCL) are crucial components of the knee joint, providing stability during dynamic movements. Despite their importance, injuries to these ligaments are common, especially in athletes. Understanding the balance between the ACL and PCL is essential for developing better treatment strategies, prevention strategies, and rehabilitation protocols.

The ACL-PCL size match has been studied in the pediatric population using coronal, sagittal, and length measurements, concluding that an intact PCL is a predictor of native ACL size [1]. In the adult population, the ACL and PCL have been analyzed in association using the same length, sagittal diameter, and coronal diameter. The length and size of the intact PCL in cases without PCL buckling are predictors of the length and size of the native ACL in adults. The use of this information to optimize graft diameter may lower the rates of ACL graft failure in the future [2].

The shape of the ACL varies along its length. In a 3T MRI study, the shape of the cross section at midsubstance 4 was an oval isthmus, which was the most narrow and well-balanced shape. It transformed into a wide band at midsubstances 1 and 5. The shape of the femoral insertion was semicircular, with its anterior side slightly straight and its posterior side convex. The tibial insertion was kidney bean-shaped [3].

The sagittal and coronal diameter cannot reflect the exact cross-sectional area of an ACL or PCL due to the complex shape of these structures. We propose a model based on 1.5T MRI for precise measurement of the cross-sectional area of the ACL and PCL. The aim of this study was to determine whether the ACL and PCL sizes are in balance when using the cross-sectional area of each structure. This information can help in choosing the most appropriate graft size in ACL or PCL reconstruction, based on the native healthy structure of the central pivot.

## Materials and methods

Study population

The current study utilizes a retrospective cohort methodology. Ethical approval, with registration number 404/17.09.2020, was obtained, and all patients provided informed consent for research participation. The study adhered to the principles outlined in the Declaration of Helsinki [4] and followed the Guidelines for Good Clinical Practice [5]. Patient eligibility criteria were established through a review of medical charts. Inclusion criteria encompassed adults over 18 years with non-contact mechanisms for knee pain, while exclusion criteria included a history of trauma, previous knee surgery, or fractures around the knee; age over 50 years at the time of evaluation; and the presence of morphologic abnormalities such as arthritis. Sixty-two patients met the inclusion criteria and were included in the data analysis process.

MRI acquisition

**Figure 1:**
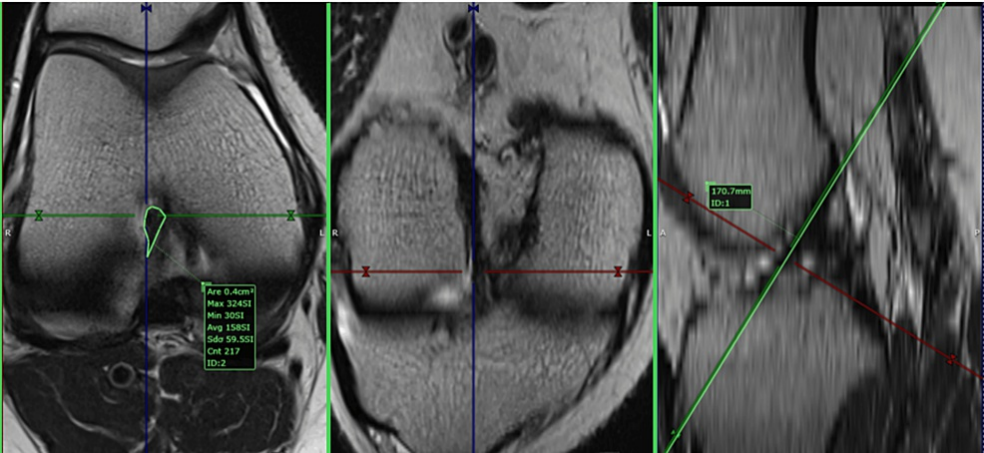
Anterior cruciate ligament (ACL) custom axial plane (aCAP), teardrop shape ACL This figure is the original work of the authors. Patient consent for the use of the image was obtained, as mentioned in the patient consent form.

**Figure 2:**
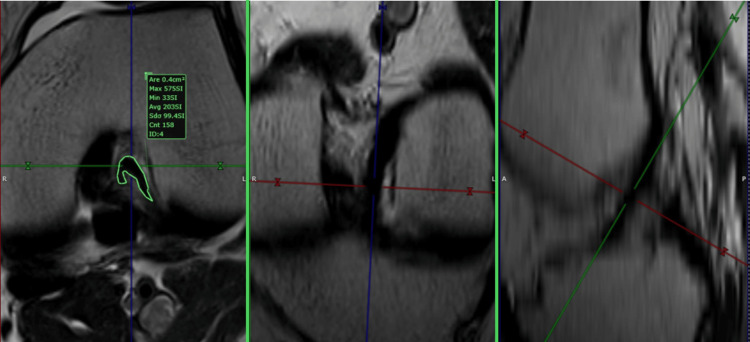
Anterior cruciate ligament (ACL) custom axial plane (aCAP), U shape ACL This figure is the original work of the authors. Patient consent for the use of the image was obtained, as mentioned in the patient consent form.

Two 1.5-T magnets (Magnetom Essenza and Magnetom Aero, Siemens Medical Solutions, Erlangen, Germany) performed the MRI examinations with similar protocols. Both provided 3 mm slices of the knee obtained using the standard three orthogonal techniques (axial, coronal, and sagittal), with a combination of fluid-sensitive sequences, either T2-weighted (T2W) non-fat-saturated (NFS) or proton density-weighted (PDW) sequences, and T1-weighted (T1W) NFS imaging. The PD-weighted sequences, with and without fat saturation, are usually the mainstay. Sometimes, the T2 NFS sequence was included as a replacement for the PD fat-saturated (FS) in the axial plane. For our study, we primarily used T2W NFS or PDW FS true axial images, employing post-processing tools to create a custom plane by applying references from the sagittal and coronal planes. The custom plane is oriented along the axis of the ACL (slightly angulated from the intercondylar roof of Blumensaat’s line) in the sagittal plane and along a line parallel to the posterior femoral condylar line in the coronal plane. As a result, the plane is perpendicular to the ACL axis and parallel to the posterior condylar line. We will call this plane the ACL custom axial plane (aCAP) (Figures 1, 2).

Using the same principle, we created a plane perpendicular to the proximal one-third of the PCL. We will call this plane the PCL custom axial plane (pCAP).

**Figure 3:**
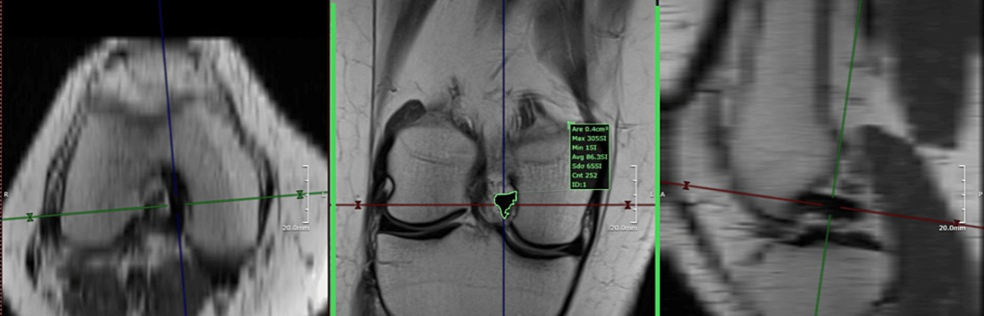
Posterior cruciate ligament (PCL) custom axial plane (pCAP), triangular shape PCL This figure is the original work of the authors. Patient consent for the use of the image was obtained, as mentioned in the patient consent form.

**Figure 4:**
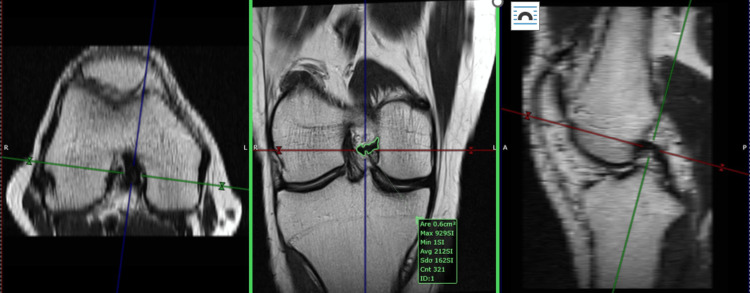
Posterior cruciate ligament (PCL) custom axial plane (pCAP), irregular shape PCL This figure is the original work of the authors. Patient consent for the use of the image was obtained, as mentioned in the patient consent form.

For the PCL, the custom plane is oriented along the axis of the PCL in the sagittal and coronal planes. As a result, the plane was axial to the PCL. The next step involved measuring the in-plane area of the PCL by outlining its contour with the closed polygon tool in the software's viewer. The measurement was done in the cranial third, in the closest possible area to the point where the PCL was not in contact with the femur. The custom plane had a slice thickness of 1 mm after post-processing (Figures 3, 4).

Variables and measurements

The in-plane area of the PCL was measured by outlining its contour with the closed polygon tool in the software's viewer. The measurement was done in the cranial third, in the closest possible area to the point where the PCL was not in contact with the femur. The custom plane (pCAP) had a slice thickness of 1 mm after post-processing.

The in-plane area of the ACL was measured in the same way, using the aCAP (ACL custom axial plane), in the cranial third. Using the aCAP, the intercondylar notch was assigned into one of three categories: A-shaped, U-shaped, and W-shaped.

Data analysis was conducted by a musculoskeletal radiologist using MRI planes tailored to the study's requirements (see Figures 1-4). These custom planes facilitated the extraction of the anterior and posterior cruciate ligaments' areas as well as the determination of the intercondylar notch shape.

Statistical analysis

Descriptive statistics, including mean and standard deviation, were employed to analyze the size of the anterior and posterior cruciate ligament cross-sectional areas (measured in cm²). Anterior and posterior cruciate ligaments' areas were described for each notch type (A, W, and U) (Table 1). To assess the normality of the data, a Shapiro-Wilk test was employed, which showed that all variables followed a non-parametric distribution (p < 0.05). Pair-wise comparisons of the ACL and PCL areas across the three notch types were investigated using the Kruskal-Wallis test with Bonferroni correction. To determine the magnitude of the difference between the ACL area in relation to the PCL area, the formula (ACL area - PCL area)/PCL area was used. The chosen significance level for hypothesis testing was set at α = 0.05. Statistical analysis was performed using IBM SPSS Statistics for Windows, Version 26 (Released 2019; IBM Corp., Armonk, New York).

## Results

**Figure 5:**
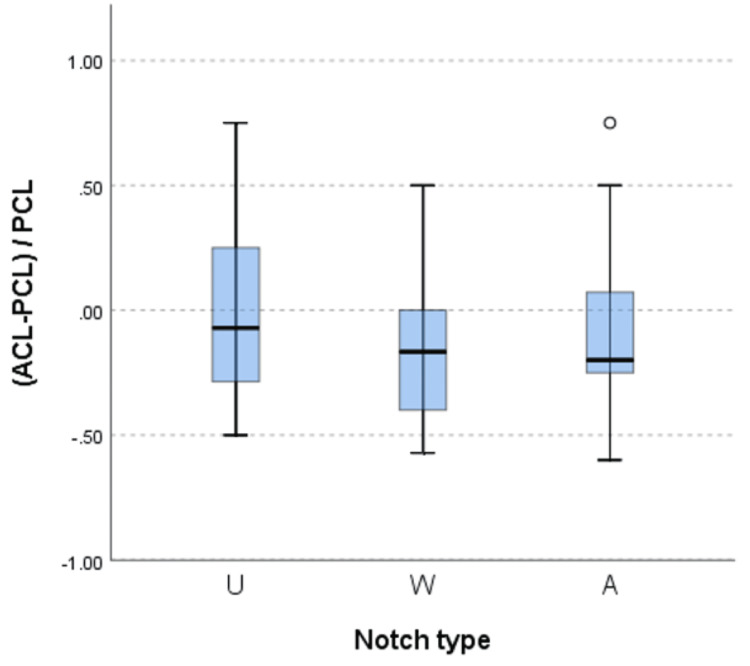
The magnitude of the area of the anterior cruciate ligament in relation to the posterior cruciate ligament across all three types of intercondylar notches. A: A-shaped notch, defined as a notch shape that narrows from the base to the apex; U: U-shaped notch, defined as a notch in which the midsection does not taper from the base; W: W-shaped notch, defined as a notch that exhibits characteristics of a U-shaped notch but also two apparent apices rather than a classic flat roof.

**Figure 6:**
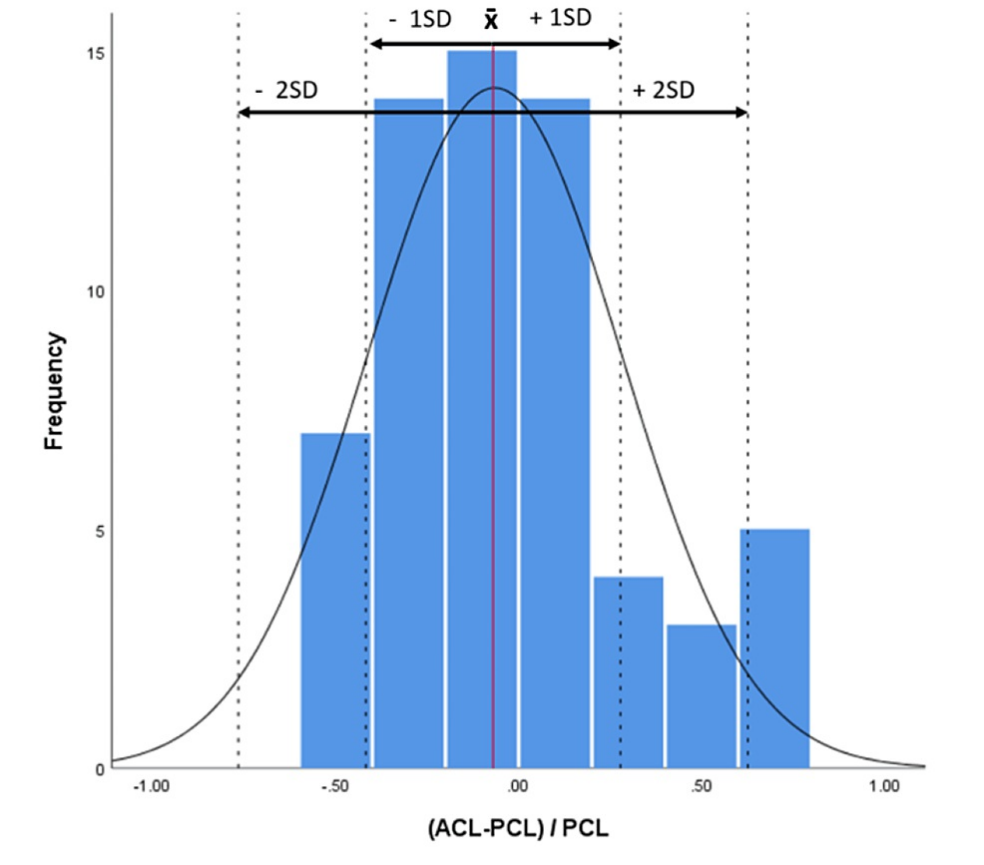
Magnitude of the difference between the ACL area in relation to the PCL area using the formula (ACL area – PCL area)/PCL area ACL: anterior cruciate ligament, PCL: posterior cruciate ligament.

A total of 31 males and 31 females were included in the study, with a mean age of 36±10 years and a mean height of 171±9 cm. The study observed three types of intercondylar notches: Type U was identified in 35% (22) of patients, Type W in 27% (17), and Type A in 37% (23) (Table 1). The median difference between the ACL and PCL areas was found to be statistically significant (p = 0.02). A significant difference in the area of the anterior cruciate ligament (ACL) was detected between Type A and Type U notches (p = 0.02), while no significant differences were found between Type A-W and Type W-U after post-hoc corrections (p > 0.05). Additionally, no significant difference was observed in the mean area of the posterior cruciate ligament (PCL) across all three notch types (p = 0.1). Furthermore, no significant difference was found between the magnitude of the ACL area relative to the PCL area across all three notch types (p = 0.5) (Figure 5), but 68% of the ACL areas were found to be within [-0.4 to 0.2] × PCL area while 95% of the ACL areas were within [-0.7 to 0.6] × PCL area (Figure 6), with negative values representing a smaller ACL area than the PCL area and positive values representing the opposite.

**Table 1:** Descriptive statistics by notch type *All measurements are reported in square centimeters (cm^2^).

Variable (cm^2^)*	Mean	SD	Min	Max
U Notch (n = 22)				
PCL area	0.55	0.14	0.4	0.8
ACL area	0.53	0.14	0.2	0.8
Notch area	1.28	0.61	0.6	3.6
W Notch (n = 17)				
PCL area	0.53	0.1	0.4	0.7
ACL area	0.43	0.09	0.3	0.6
Notch area	0.39	0.17	0.15	0.81
A Notch (n = 23)				
PCL area	0.48	0.1	0.3	0.7
ACL area	0.43	0.15	0.2	0.8
Notch area	0.49	0.18	0.15	0.95

## Discussion

The three types of notch geometry (A, W, and U) have been described by van Eck et al. [6]. This geometry has been accepted and used for various purposes. Using this geometry, we provided evidence that the ACL sectional area is correlated with the anterior intercondylar notch area in a previous study [6-14].

The cross-sectional area varies in the three notch types for ACL and PCL. A significant difference in the ACL area is observed between type A and type U notchs. Regarding the PCL, no significant difference was found in the measured area between the three types of notches. The median difference between the ACL and PCL areas was found to be statistically significant.

When examining the ACL-PCL balance, using the ACL-PCL/PCL formula, no significant difference was found between the magnitude of the ACL area relative to the PCL area across all three notch types (Figure 5), but 68% of the ACL areas were found to be within [-0.4 to 0.2] × PCL area while 95% of the ACL areas were within [-0.7 to 0.6] × PCL area (Figure 6), with negative values representing a smaller ACL area than the PCL area and positive values representing the opposite.

This study contributes to the existing literature by providing a greater understanding of the real cross-sectional area relationship between ACL and PCL. Although other studies have focused on this aspect, to our knowledge, this is the first study to analyze the ACL-PCL balance using the cross-sectional area. The statistical findings should be interpreted in the clinical/surgical context. Although we did not find a statistically significant match between the two structures, a larger series might be able to prove such a correlation.

A recent study showed a significant difference in the volumes of the intercondylar notch and the ACL between patients with a ruptured PCL and control patients. Patients with a PCL rupture have smaller intercondylar notch volumes and smaller ACL volumes. There were no significant differences in the bicondylar width, notch width, and notch width index. In the control patients, a significant correlation between the volume of the PCL and the volume of the ACL was found (0.673, p < 0.001) [15]. This volume correlation, although using a different measurement tool, supports the same principle: an ACL-PCL balance in the knee.

Another recent study, done on pediatric patients, found that correlations exist between the ACL, PCL, and patellar tendon in normal pediatric knees, and that equations can be created that predict ACL size based on the intact PCL and patellar tendon. Although multiple anatomical measurements were made and can be predicted using these equations, the most clinically relevant equations are likely those that predict ACL length, ACL mid-substance thickness, and ACL mid-substance width [16]. Although this study is a pediatric one, the authors included 140 MRIs of patients aged 8-12 years, as well as 200 male patients aged 13-18 years and 200 female patients aged 13-18 years. Many of these patients must have had skeletal maturity at the age of the MRI exam. The same principle stands out from this study: an ACL-PCL balance in the knee.

Another study proved that cruciate ligaments are hypertrophied in weightlifters similarly to other soft tissue structures. The authors concluded that currently, they cannot predict the role this may play for the athletes' knees in the future but impingement between ligaments is possible. On the other hand, overgrown cruciate ligaments might support knee joint stability and have a protective role in ACL injury, especially in patients with a defined risk factor such as a steeper lateral tibial plateau slope [17]. The same principle is suggested: an ACL-PCL balance in the knee.

We think that the knowledge of this phenomenon may be helpful for many doctors treating specific conditions such as ACL or PCL lesions.

Limitations of the study

**Figure 7:**
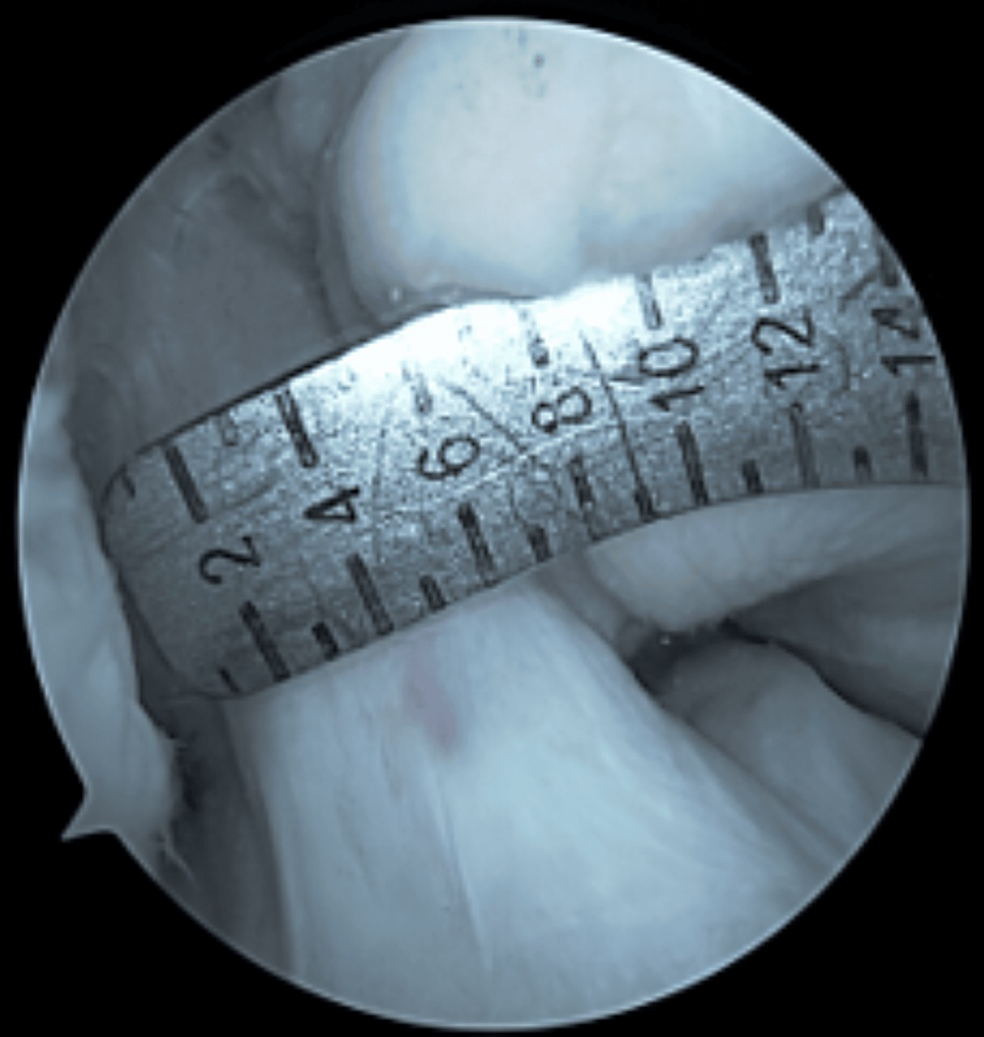
Native ACL: 7 mm diameter (0.3843 sq. cm area) Native anterior cruciate ligament (ACL): non-operated, non-injured ACL. This figure is the original work of the authors. Patient consent for the use of the image was obtained, as mentioned in the patient consent form.

**Figure 8:**
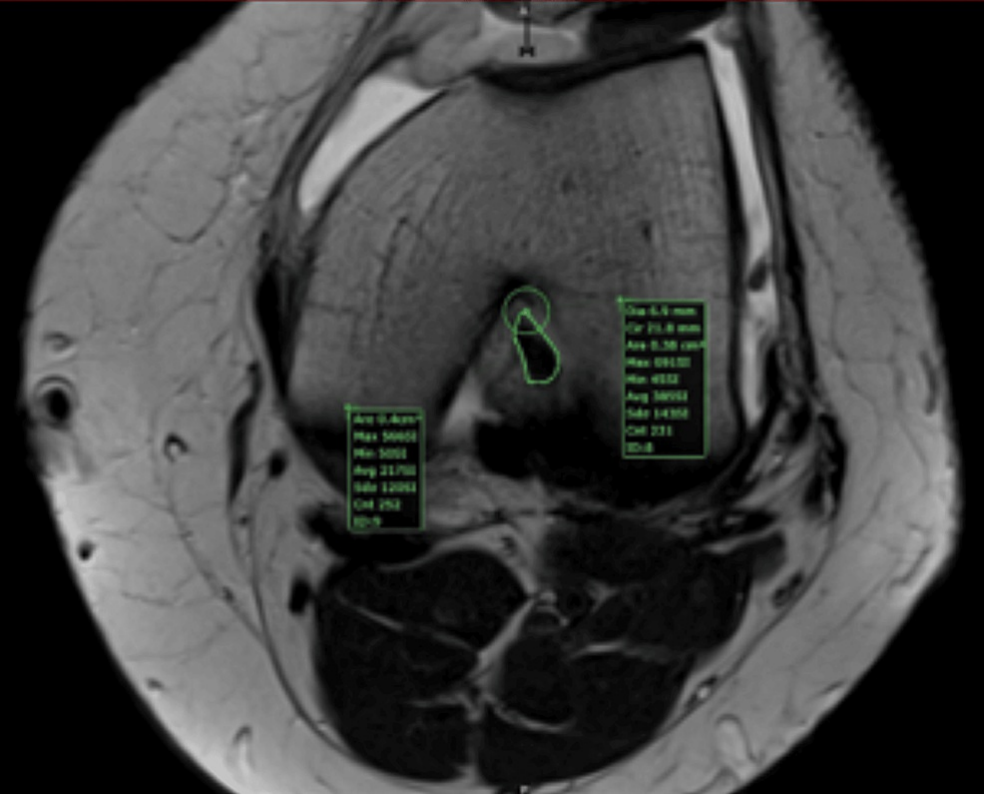
MRI measurement of the same anterior cruciate ligament (ACL) presented in Figure 7 (0.38 sq. cm area) This figure is the original work of the authors. Patient consent for the use of the image was obtained, as mentioned in the patient consent form.

Our study is a cross-sectional study with retrospective inclusion and lacks interobserver agreement. The measurement technique is not validated. To compensate for this aspect, we compared the intra-operative dimension of a native ACL with the MRI measurement (Figures 7, 8) and found a precise match. Because of the lack of a series, we could not validate the method.

The second limitation of the study is the statistical power of the series. We believe a stronger correlation could be found if the two structures were analyzed in a larger series for each notch type. The third limitation of this study is the racial diversity. All of our patients are Caucasian. The fourth limitation arises from the fact that most of the PCLs in ACL-deficient knees are buckled. This would hamper the preoperative MRI measurement of the PCL. ACL graft size is different from the actual ACL size post-epithelialization. Thus, judging the graft size according to the intact PCL might be erroneous.

In 68% of the cases, the ACL is no less than 60% of the PCL in area, and no more than 120%. For example, if the PCL area is 0.79 cm² and the measured structure is round (during a reconstruction a hamstring graft is round), the diameter is 10 mm. A native ACL is, in 68% of the cases, no less than 7.7 mm, and no more than 10.9 mm. The size of ACL and PCL in healthy individuals also depends on other factors like synergistic and antagonistic muscle activities, occupation, and the hip-knee-ankle axis. Standardizing this correlation for surgical use could prove erroneous.

## Conclusions

The balance between ACL and PCL ligaments impacts the tibiofemoral biomechanics. Each structure is the opposite of the other in terms of anteroposterior translation and rotation. The ACL-PCL size correlation helps in understanding the balance of the central pivot of the knee. In our study, in more than half of the studied patients, the ACL is no less than 60% of the PCL in area (7.7 mm), and no more than 120% (10.0 mm).
